# Enhancing Intra‐Specific Population Genetic Studies of Bottlenose Dolphins ( *Tursiops truncatus* ) in Irish Waters by Integrating Least Invasive Sampling and Environmental DNA (eDNA)

**DOI:** 10.1002/ece3.74035

**Published:** 2026-08-14

**Authors:** Lorenzo De Bonis, Bogna Griffin, Sean O'Callaghan, Mags Daly, Jade Maes, Simon Berrow, Luca Mirimin

**Affiliations:** ^1^ University College Cork Cork Ireland; ^2^ Atlantic Technological University Galway Ireland; ^3^ Marine Institute Oranmore Ireland; ^4^ Irish Whale and Dolphin Group Kilrush Ireland

## Abstract

Population genetic studies provide important data for evidence‐based conservation and management of marine mammal species. Obtaining tissue and genetic material from marine mammals is challenging, often leading to limited sample sizes, however emerging least‐invasive sampling methods, such as extra‐organismal DNA (e.g., blows) and environmental DNA (eDNA) (e.g., water), have the potential to provide additional samples for intra‐specific genetic studies. Thus, this study aimed at further investigating population genetic structure of Irish bottlenose dolphins ( *Tursiops truncatus* ) by corroborating samples sourced with direct methods (e.g., biopsies of free‐ranging dolphins and tissue from dead dolphins) (*n* = 67) with samples collected using least invasive indirect methods (eDNA isolated from water, or extraorganismal DNA from blows, faeces, and sloughed skin, collected opportunistically in proximity to individuals) (*n* = 52). Using 15 microsatellite markers, a 540 bp portion of mitochondrial DNA (mtDNA) control region and nuclear sex markers, intra‐specific genetic information was obtained from samples sourced with indirect methods, including previously‐known mtDNA haplotypes recovered from 11 samples (21% success rate) and consistent microsatellite genotypes from 5 of 15 markers, sufficient for assigning individuals to population of origin. This work led to the most comprehensive population genetic study of Irish bottlenose dolphins to date (140 individuals), confirming the presence of three genetically distinct populations, namely an offshore ecotype and two coastal resident populations (the Shannon Estuary and the Connemara‐Mayo‐Donegal population). Importantly, findings from this study provide corroborative evidence that Shannon dolphins use Brandon Bay (Co. Kerry) as a key foraging area, which is outside the current Shannon Estuary Special Area of Conservation (SAC) boundary, hence advocating a revision of such boundaries. Additionally, this study included samples from a number of Irish “solitary” dolphins, which were genetically assigned to the offshore ecotype, further showcasing least‐invasive sampling methods, including eDNA, as a promising tools for the study of intra‐specific genetic variation.

## Graphical abstract

Three populations of bottlenose dolphins are found in Irish waters. This study used direct sampling methods to expand the sample size and confirm the distinctiveness of these populations, including a range expansion outside of the Shannon Estuary SAC. Environmental DNA sampling was successful at securing intraspecific genetic data from solitary dolphins revealing their population of origin.

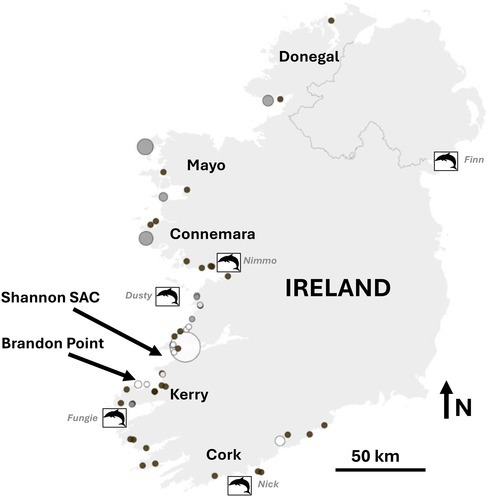

## 1 Introduction

Common bottlenose dolphins ( *Tursiops truncatus* , Montagu, 1821) occur year‐round in Irish coastal and offshore waters (O'Brien et al. 2009; Oudejans et al. 2015; Barker and Berrow 2016; Rogan et al. 2018). Bottlenose dolphins are top predators and can have a major impact on the ecosystem they are part of (Heithaus et al. 2008), but are also sentinels of an ecosystem's response to climatic variation and anthropological disturbance (Young et al. 2015; Hazen et al. 2019; López and Shirai 2008). In Irish coastal waters, bottlenose dolphins require strict protection and are entitled to formal protection of a representative range of their habitats as Special Areas of Conservation (SAC) under the EU Habitats Directive. The Lower River Shannon SAC was designated in 2000 to provide protection to a small, genetically discrete population of bottlenose dolphins (map available in Figure S1a). This population has been studied since 1993 and shows a high level of residency all year round (Berrow et al. 1996; Ingram 2000), as well as genetic distinctiveness (Mirimin et al. 2011). Their abundance was last estimated to be 116 ± 9 (95% CI 103–122) individuals during the summer sampling period (Berrow et al. 2012) and it is believed that this population has been relatively stable over the last 25 years (Blázquez et al. 2020). The West Connacht Coast SAC was designated in 2013 to provide protection for a second genetically distinct population, which is known to range throughout Irish coastal waters (O'Brien et al. 2009; Mirimin et al. 2011) (map available in Figure S1b). Abundance and residency patterns of this population have proven to be much harder to estimate. In 2009, an abundance estimate of 171 ± 48 (95% CI 100–294) was derived from dedicated survey data collected during surveys of north Connemara (Co. Galway) waters (Ingram et al. 2009); however, it was apparent that the animals were ranging well beyond this area with matches of individuals as far apart as Co. Cork Co. Donegal (Ingram and Rogan 2003) and even Scotland and the UK (Robinson et al. 2012), with the most recent abundance estimate (Berrow et al. 2025) of 201 ± 27 (95% CI 148–254). Besides these two coastal populations, the hypothesis of a third distinct population has been supported by genetic (Mirimin et al. 2011; Nykänen, Kaschner, et al. 2019; Nykänen, Louis, et al. 2019), behavioural (Oudejans et al. 2015) and morphological evidence (Hersh and Duffield 1990), which reflects the existence of two ecotypes (coastal vs. pelagic) as is the case in the northeast Atlantic (Duffield et al. 1983; Mead and Potter 1995; Louis et al. 2014; Costa et al. 2016; Fruet et al. 2017). As a consequence of such mounting evidence, in 2024, bottlenose dolphins were added as qualifying interests to five existing SACs including three offshore sites (Porcupine Bank Canyon SAC, Southwest Porcupine Bank SAC and Belgica Mound Province SAC) (detailed maps provided in Figure S1b).

Bottlenose dolphins are generally highly sociable animals and rely on cooperation within and among social units, however occasionally individuals have also been observed to become socially isolated and have taken long‐term residency often in geographically confined coastal areas; in fact, at least eight such solitary dolphins have been recorded in Ireland (Nunny and Simmonds 2019; Goodwin and Dodds 2008). These dolphins largely do not interact with other bottlenose dolphins and live a relatively solitary life, but tend to seek out human interactions (Nunny and Simmonds 2019). These include Fungie, a male individual that was first reported in 1983 in Dingle Harbour (Co. Kerry), where he was the longest recorded solitary cetacean globally (37 years) before he disappeared in October 2020 (Nunny and Simmonds 2019, Goodwin and Dodds 2008). The second longest recorded solitary individual in Ireland is a female (Dusty) who was first recorded in 2000 in Doolin (Co. Clare) and is known to occur from north Clare to the Aran Islands (Co. Galway), where she has regularly interacted with swimmers (Nunny and Simmonds 2019; Goodwin and Dodds 2008). More recently, two other solitary dolphins have been recorded at the mouth of the River Corrib near Galway City (Co. Galway) and at Greenore (Co. Louth), named Nimmo and Finn, respectively. Finally, a transient sociable‐solitary dolphin (Nick), who is known to have moved between the south coast of Ireland and the southwest coast of England in 2021, was found dead in Cork harbour after a fatal boat strike (propeller marks were found on the carcass). These resident coastal dolphins have been observed interacting very regularly with people and have contributed significantly to the local dolphin watching industry (Hoyt 2001); however, surprisingly little is known about such individuals, including their population of origin.

Multiple genetic studies on bottlenose dolphins have been carried out in Ireland (Mirimin et al. 2011; Nykänen et al. 2018) and adjacent Northeast Atlantic waters (Louis et al. 2014, 2021; Dinis et al. 2016; Nykänen, Kaschner, et al. 2019; Nykänen, Louis, et al. 2019). However, while these studies have significantly advanced our understanding of population structure, the numbers and location of sampled individuals are often hampered by opportunistic sampling strategies and/or challenging environments (such as the difficulties of obtaining biopsy samples in offshore waters). To address current knowledge gaps, it is vital to expand the number and range of samples, especially from areas that are still underrepresented. In recent years, the advancement of molecular methods has enabled the collection of genetic material using least invasive methods, such as epidermal cells in blows or faeces or even as traces of environmental DNA (eDNA) in water (Parsons et al. 2018, Sigsgaard et al. 2016, 2020, Weitemier et al. 2021, Dugal et al. 2022, Parsons et al. 2024). In fact, virtually all organisms shed biological material in the surrounding environment, which can be used to isolate extraorganismal DNA originating from cells, organelles, or eDNA adsorbed to particles or dissolved in water (Rodriguez‐Ezpeleta et al. 2021). Least invasive methods provide advantages over direct methods such as biopsies as they typically do not require specific permission (as they cause less stress to the sampled individuals) and can be performed by non‐specialists, following some basic training. Samples collected in close proximity to individuals of interest (such as sampling water from a fluke print or blow sampling) can enable the collection of genetic material from that single targeted individual (Frère et al. 2010; Székely et al. 2021; Arranz et al. 2024; Bennington et al. 2024; O'Mahony et al. 2024; Blázquez et al. submitted). While obtaining high quality genetic data (such as nuclear single nucleotide polymorphisms [SNPs] and microsatellites) from environmental samples is challenging, an increasing number of studies have shown the ability to detect population specific variants by means of DNA sequencing of short mitochondrial DNA (mtDNA) fragments (Baker et al. 2023; Bennington et al. 2024; Parsons et al. 2024).

Thus, this study aims to expand our knowledge of bottlenose dolphin population structure in Irish waters using the largest nuclear and mitochondrial genetic data set of Irish dolphins yet analysed. Specifically, besides the inclusion of additional samples from dead strandings and biopsies, the use of least‐invasive sampling approaches was explored as a means to obtain additional genetic information at the intra‐specific level with the goal of investigating a potential home range expansion of coastal resident populations and to discern the population of origin of charismatic solitary social dolphins found in Irish waters.

## 2 Materials and Methods

### 2.1 Sample Acquisition and DNA Isolation

**FIGURE 1:**
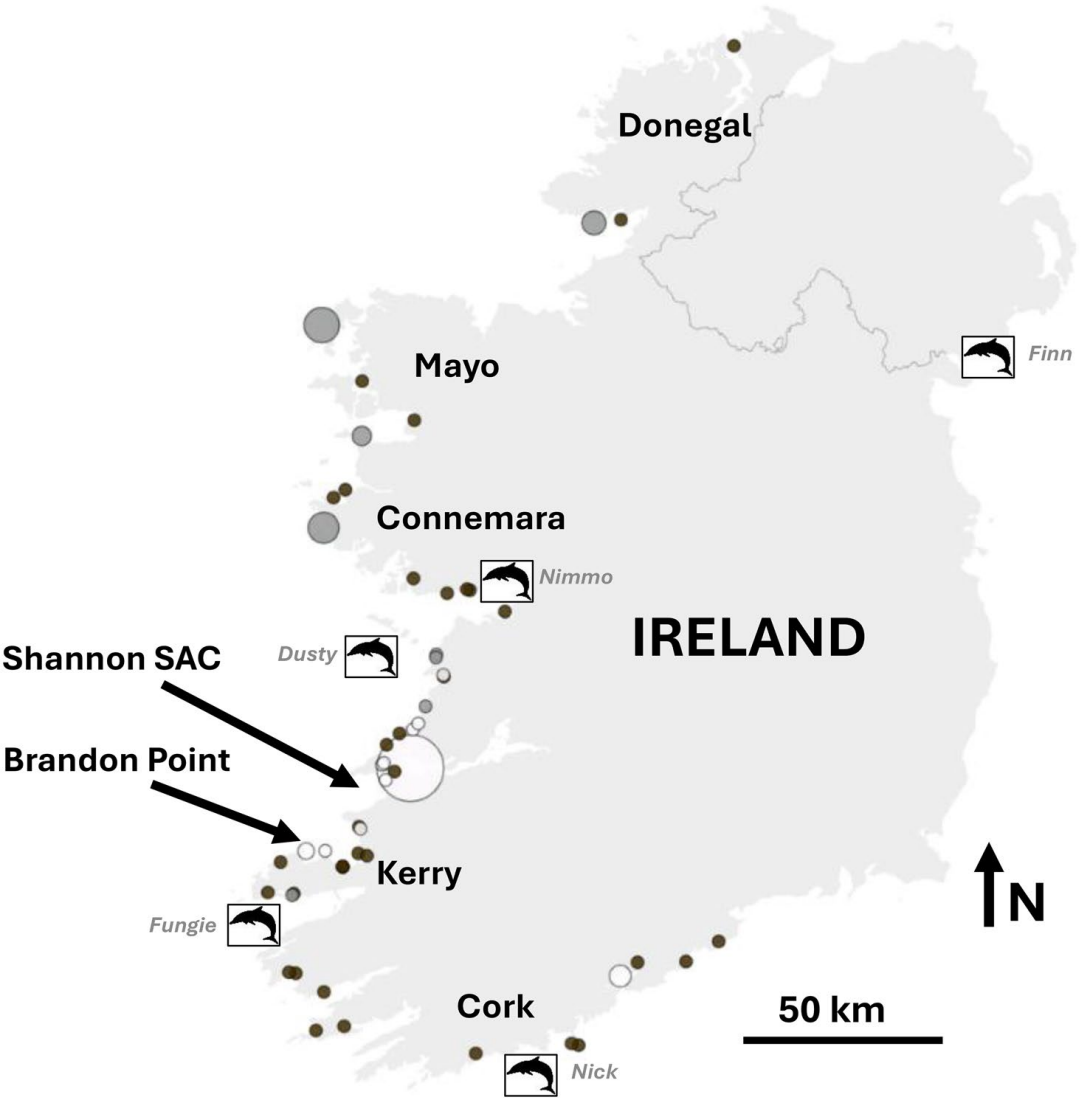
Map showing all sampling locations and sample types across the three Irish populations of bottlenose dolphins. Colours reflect genotypes as illustrated in Figure 2 (Black: offshore/strandings; White: Shannon; Grey: North‐West (Connemara‐Mayo‐Donegal)). Size of circles is proportional to sample size. The dolphin symbols indicates approximate locations of solitary dolphins and their respective names in grey. Further details on exact location of sampling is provided in Table S1. Detailed maps of SACs can be found in Figure S1.

The samples used in this study derive from three different sources: skin tissue from stranded individuals (*n* = 48), biopsy of free‐ranging dolphins (*n* = 19) and least invasive sampling (*n* = 52) (Figure 1) (further details available in Table S1). Samples from stranded individuals were collected along the Irish coastline between 2000 and 2021. Samples from free‐ranging bottlenose dolphins were collected using 20–25 mm tips delivered by a Barnett Crossbow during dedicated surveys between 2011 and 2014 carried out under licence by the Irish Whale and Dolphin Group (licence numbers C104/2011, C195/2012, C011/2013, C006/2014, C060/2015). Tissue samples from live and stranded dolphins were preserved in 96%–100% ethanol and DNA was extracted from approximately 25 mg of tissue using the DNeasy Blood & Tissue kit (QIAGEN). A total of 26 seawater samples and nine blow samples were collected opportunistically from RV *Celtic Mist* during a bottlenose dolphin survey between the 18 and 24 July 2021 within the Brandon and Tralee Bays Co Kerry and in the Shannon Estuary (additional info available in Table S1). At the time of all sampling, no other cetaceans aside from bottlenose dolphins were present, although sightings of common dolphins ( *Delphinus delphis* ) and minke whales (*Balaenoptera acurostrata*) were made elsewhere during the survey. Samples of 5 L of water were filtered directly from the sea surface, in proximity to free‐ranging dolphins, using tubing weighted with a steel weight suspended from the moving vessel. No unusual behavioural reactions were noted from dolphins during water sampling. Water was pumped when close to dolphins (while bow riding or adjacent to the vessel) onto glass fibre filters (47 mm AP25, 2 μm pore size; Millipore, Billerica, USA) housed in an in‐line filter holder, using a portable peristaltic pump (Masterflex E/S portable sampler, Cole‐Parmer, Vermon Hills, IL, USA). To avoid cross‐sample contamination, all equipment used for filtering (in‐line holder and tubing) was placed in a 10% sodium thiosulfate solution between samples and rinsed with tap water. Blow samples were collected by placing a glass fibre filter (same as above) inside a petri dish mounted onto a telescopic pole positioned above the dolphin's blowhole while the animals were bow‐riding. All filters were folded in half and placed in individually labelled zip lock bags and stored in an onboard freezer at −24°C. Filters were then transported to the laboratory in a cooler bag with ice blocks and stored at −80°C until further processing. Outside of the dedicated bottlenose dolphin survey aboard the RV *Celtic Mist*, a total of 12 additional samples of various types (two shed skin, two faeces, one wetsuit swab and 12 5 L seawater samples) were collected in the presence of solitary social dolphins. Water samples were processed as described above and additional information on these samples can be found in Table S1. For all least‐invasive samples, DNA was extracted from one half of the filter following a modified DNeasy Blood & Tissue kit (QIAGEN) (as described in Tibone et al. 2024). All DNA extractions were performed in a PCR‐free laboratory in a laminar flow BSC2 cabinet. Quantity of DNA extracts was measured using a Nanodrop One spectrophotometer (Thermo Scientific).

### 2.2 Molecular Sexing and Nuclear Microsatellites Genotyping

Prior to conducting any analysis in vitro, in silico Primer‐BLAST searches using default parameters (Ye et al. 2012) were carried out to assess the potential for cross‐species amplification (e.g., false positives) of all markers (primer pairs) used in the present study. This was done because most markers were originally conceived for amplification of target DNA from genomic DNA isolated from biological material originating from a single individual (e.g., tissue), whereas this study also included some environmental samples, which are likely to contain DNA from multiple taxa and may lead to non‐specific amplification. Besides in silico tests, amplicon size, expected results (sex, where possible) and DNA sequence data (mtDNA only) were used to assess whether results could be trustable. Additionally, genotyping errors were estimated by repeating a number of samples (detailed below), including 12 individuals that were already used in a previous study (Mirimin et al. 2011).

Nuclear molecular markers located in genes located in sex chromosomes (ZFX & SRY) were used to identify the sex of individuals from collected samples, following the method described in Rosel (2003).

A total of 15 nuclear microsatellite loci used in the previous largest study on Irish bottlenose dolphins (Mirimin et al. 2011) were selected for nuclear multi‐locus genotyping. The 15 microsatellite primer pairs were run in multiplex in three different sets, each set targeting five loci (see further details in Table S2), with the forward marker labelled with a spectrally distinct fluorophore and in the presence of negative (no template) controls in each run. PCR products were visualised on 2% agarose gels stained with SYBR Safe to check for amplification, then diluted 1/10 prior to resolving the size of amplicons by capillary electrophoresis by comparison to a GeneScan 500 LIZ dye Size Standard on a 3730xl DNA Analyzer (Applied Biosystems). To investigate possible disagreement in allele amplification between different PCR runs, 20% of samples originating from tissue and all DNA extracts from samples collected with least‐invasive methods were run twice. Amplicon/allele sizes were resolved using the software GeneMapper v4.1 (Applied Biosystems) and each trace file was inspected by eye to identify microsatellite‐like patterns. For the latter, a conservative approach was used whereby allele variants detected by least‐invasive sampling were only retained if previously identified (at a frequency of 0.05 or higher) in the variant set obtained with high quality DNA from tissue samples. Multi‐locus genotypes derived from least‐invasive samples collected in the presence of the same individual were merged into one composite genotype by including only allelic variants that were in agreement among samples and by discarding any mismatching data. For environmental samples collected in the presence of multiple individuals and showing more than two putative alleles for the same sample, the alleles with the highest peaks were chosen. As done with the mtDNA data, the genotype data produced in this study was merged with the genotype data of the individuals analysed in Mirimin et al. (2011), increasing the total number of individuals analysed for population structure analysis using nuclear markers from 85 to 127. This was possible since the set of markers used between the two studies is identical and the studies included a subset of shared common samples of 12 individuals, which also acted as a quality control step in the present study. To check for duplicate samples outside of the known ones, the function “Multilocus Matches” of GenAlEx 6.5 (Peakall and Smouse 2006) was used to compare multi‐locus genotypes between all pairs of dolphins. The function “Multilocus Prob. Identity” (Waits et al. 2001) provided an estimate of the average probability that two unrelated individuals drawn from the same population will have the same multi‐locus genotype. The software STRUCTURE Version 2.3.4 (Pritchard et al. 2000) was used to investigate population structure without prior information on sampling location. To investigate the minimum number of successfully genotyped loci to correctly assign a sample to its cluster, a simulation of 140 separate STRUCTURE runs was carried out, using 100 samples that were successfully genotyped at all 15 markers. In each run, a random combination of markers had missing data across all samples, 10 combinations for each possible number of no‐data markers (1–14), and the result was then compared to the run with all 15 markers. To converge to the best number of clusters (*K*), 10 Markov Chains Monte Carlo (MCMC) runs were carried out for each of six *K*‐values (1–6), using the admixture model and correlated allele frequencies among putative populations. The parameters chosen for the runs where 100,000 (burn in) and 1,000,000 MCMC repetitions. Results from all 10 runs were summarised and inspected using STRUCTURE HARVESTER Version 0.6.94, using the Evanno's method for identifying the number of K that best fits the data (Earl and von Holdt 2012). Once the best *K* number was determined, each individual/sample was assigned to its putative population by inspecting the proportion of membership to each of the inferred cluster (assigning to the cluster with the highest value), as observed in the final run. Consequently, for each population (as identified by STRUCTURE) the following tests were performed. Micro‐Checker 2.2.3 (Van Oosterhout et al. 2004) was used to test for presence of null alleles, allelic dropout and potential scoring errors due to stuttering, selecting the Bonferroni adjusted 95% confidence interval option (1000 simulations). Using ARLEQUIN 3.5 (Excoffier and Lischer 2010) and GenAlEx 6.5, possible linkage disequilibrium between all pairs of loci was tested. Expected and observed heterozygosity and Hardy–Weinberg equilibrium tests were carried out to detect possible departure from equilibrium. Weir and Cockerham (1984)'s theta (*F*st) was estimated per overall and for each pair of populations, with significance (*p* values) estimated after 1000 randomizations with an indicative adjusted nominal level (5%) of 0.017 using FSTAT 2.9.4 (Goudet 2001).

### 2.3 Mitochondrial DNA Sequencing

Presence of bottlenose dolphin mitochondrial DNA (mtDNA) was investigated using primers Dlp1.5 5′‐TCACCCAAAGCTGRARTTCTA‐3′ (Baker et al. 1998) and Dlp8G 5′‐GGAGTACTATGTCCTGTAACCA‐3′ (Dalebout et al. 2005), targeting a portion of the mtDNA control region, following protocols as described in Mirimin et al. (2011). Potential cross‐sample contamination during the PCR step was assessed in the presence of negative (no template) controls and successful PCR amplification was assessed by agarose gel electrophoresis. Subsequently, Sanger DNA sequencing was performed using both primers by a third party sequencing provider (GENEWIZ, Azenta Life Sciences, Germany); including clean‐up by magnetic bead‐based high‐throughput purification, BigDye v3 chemistry and using an ABI3730xl DNA Analyser (Applied Biosystems). Quality of DNA sequencing results (trace files) were inspected by eye and alignments were performed using the ClustalW algorithm as implemented in the Molecular Evolutionary Genetic Analysis (MEGA) software version 11 (Tamura et al. 2021). A BLAST was performed to confirm that DNA sequences obtained in this study were of the correct target taxon and mtDNA region. In addition to DNA sequence data obtained in the present study, previously published data from Mirimin et al. (2011) were included in subsequent analysis to increase sample size and geographic coverage of bottlenose dolphins from Irish waters (Genbank accession number HQ634245–HQ634258). The number of haplotypes, haplotype diversity, nucleotide diversity and tests of neutrality (Excoffier and Lischer 2010), as well as an Analysis of Molecular Variance (AMOVA) were run together with a pairwise comparison test in order to estimate the degree of population differentiation using the software Arlequin version 3.5.2.2 (Excoffier and Lischer 2010).

## 3 Results

### 3.1 DNA Yield and Marker Usability Across Sample Types and Molecular Sex Determination

Total amount of extracted DNA varied substantially between and within different sample types, with eDNA and other extraorganismal DNA sample types showing lower concentrations than tissue samples (live biopsy or dead stranded) (Table 1) (see also data in Table S1). As for “usability” of extracted DNA, as expected, tissue samples showed the highest yield in terms of genetic data across mtDNA and nuclear (sex and microsatellite) markers, whereas eDNA and extraorganismal samples shower substantially lower (but not negligible) proportions of usable data (Table 1). Tests for primer specificity carried out in silico (Primer‐BLAST) showed that the used markers demonstrated some level of cross‐sample amplification (albeit within some marine mammal species; results in Table S2), hence urging caution when collecting environmental samples in the presence of multiple species, as indicated in the original publications (e.g., Coughlan et al. 2006). Nonetheless, given the circumstances of sampling (e.g., proximity to target individual) and the availability of samples where expected results were known, such as results of molecular sexing from least invasive sampling confirming sex of known individuals (e.g., solitary dolphins), we expect that results presented from least invasive methods show an acceptable level of reliability.

**TABLE 1:** Mean (± standard deviation) of DNA extracts yield (quantity) and molecular marker success rates by sample type.

Sample type	DNA quantity (ng/μL)	mtDNA sequencing success	Sex determination success
Tissue‐live‐biopsy (*n* = 19)	65.0 ± 21.7	19/19 (100%)	19/19 (100%)
Tissue‐dead‐stranding (*n* = 48)	47.5 ± 45.2	43/48 (90%)	38/48 (79%)
eDNA‐Seawater (*n* = 38)	34.1 ± 24.1	6/38 (16%)	45/38 (42%)
Extraorganismal DNA‐blow skin (*n* = 9)	33.4 ± 21.5	2/9 (22%)	1/9 (11%)
Extraorganismal DNA‐faeces (*n* = 2)	26.0 ± 26.5	2/2 (100%)	2/2 (100%)
Extraorganismal DNA‐sloughed skin (*n* = 2)	1.4 ± 0.3	0/2 (0%)	1/2 (50%)
Extraorganismal DNA‐wetsuit swab (*n* = 1)	3.7	0/1 (0%)	1/1 (100%)

Four eDNA sea water samples collected in the presence of the solitary dolphin “Dusty” were merged in one genotype, and the same was done for the five and six eDNA sea water samples collected in the presence of solitary dolphins “Fin” and “Nimmo”, respectively. While sample size is limited, samples from sloughed skin and faeces provided usable data as well as eDNA from sea water samples, despite some level of discordance (see also data in Table S3), thus individual genotypes were created and used for subsequent population assignment analyses.

The probability (PI) that two individuals shared the same genotype over the 15 microsatellite markers was calculated at 9.21^−15^ for unrelated individuals and 3.02^−6^ for siblings. Subsequently, a number of identical genotypes were identified across the tissue sample set, allowing the identification of three duplicate individuals: one stranded dolphin sampled twice and two individuals biopsied twice.

Sex ratio was approximately 1:1 in live free‐ranging (biopsied) individuals (*f* = 9, *m* = 10), while it was biased towards males in dead stranded individuals (*f* = 13, *m* = 25). As expected, nuclear microsatellite genotyping was very successful for DNA from tissue samples (ranging from 11 to 15 (out of 15) markers per sample), whereas a lower success rate was observed in samples collected using least invasive methods (ranging from 5 to 14 markers per sample; see also data in Table S3). Genotyping errors estimated from duplicate runs were also low for DNA from tissue samples (1.3%), and slightly higher for least invasive samples (2.7%).

### 3.2 Nuclear Microsatellite Markers Genotyping

For each of the genetically distinct populations, no linkage was identified between pairs of microsatellite loci and no sign of allelic dropout or null alleles was detected. The total number of distinct individuals included in this population structure analysis was 129, including 85 from the Mirimin et al. (2011) study and 44 new individuals obtained for the present study. Levels of genetic diversity were similar to those found in Mirimin et al. (2011), with the offshore population presenting the highest degree of genetic diversity (see Table S4).

**FIGURE 2:**
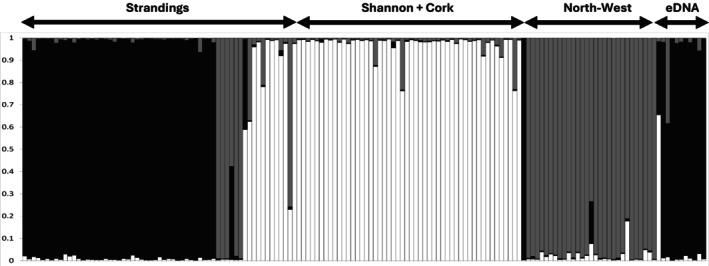
Proportion of membership of each individual (represented by the coloured vertical bar) to the three clusters (black, white and grey). Samples depicted as ‘eDNA’ include the four solitary dolphins followed by the seven sea water samples collected from the RV *Celtic Mist* (six sea water samples and one blow).

Results of the STRUCTURE simulation runs indicated that an individual has an increasing chance of being assigned to the correct population as the number of markers increases from 1 to 15 (see graph in Figure S2). The curve starts to reach a plateau at 7 markers, where there would be a 95% (±1.46) chance of correct assignment. Ultimately, all individuals included in the STRUCTURE runs were successfully genotyped at least at 7 loci (after removing disagreement alleles and potential false alleles), except for solitary dolphins Nimmo and Fin that were genotyped at 5 loci. Results from STRUCTURE harvester showed the highest delta *K* value for *K* = 2 (suggestive of a first level of sub‐structuring), but an *L*(*K*) indicating that the dataset was best explained by a three cluster (*K*) model (see graph in Figure S3), reflecting a hierarchical population structuring pattern and confirming the presence of three distinctive populations in Irish waters (further supported by *F*_st_ results below). The proportion of membership of each individual genotype to each of the three clusters is shown in Figure 2. Cluster one (blue) is composed of stranded dolphins or by environmental samples collected in the presence of solitary dolphins. This includes individuals assigned to the putative offshore populations (as hypothesised by Mirimin et al. 2011), which still remains the most plausible explanation as no other coastal/resident populations of bottlenose dolphins are known to occur in Irish waters. Cluster two (red) is represented entirely by animals biopsied, stranded, or eDNA from sea water samples within 70 km of the Shannon Estuary and four individuals biopsied in Cork harbour. Cluster three (green) is comprised for the major part (30 out of 35) by dolphins biopsied either in Connemara, Mayo or Donegal (Northwest Ireland, NW). Four animals genetically belonging to the Connemara‐Mayo‐Donegal (NW) cluster were found stranded in Co. Kerry (*n* = 2) and Co. Clare (*n* = 2), while the remaining putative NW individual was biopsied in the Shannon Estuary. Three out of the four solitary social dolphins (Dusty, Fin and Nimmo) were assigned to the offshore population while the remaining individual (Fungie) had an intermediate genotype (Shannon/offshore). The fifth solitary social dolphin (Nick), whose DNA was isolated from tissue sampled after being found dead in Cork harbour, was also assigned to the offshore population. *F*_st_ estimates for population differentiation showed an overall *F*_st_ of 0.127 and pairwise *F*_st_ values of 0.125, 0.085 and 0.166, for offshore‐Shannon, offshore‐NW and Shannon‐NW, respectively; all of which were significant at the 0.001 level.

### 3.3 mtDNA Sequencing Results

Out of the 52 samples collected using least invasive methods, mtDNA sequences matching to *Tursiops truncatus* were obtained from 11 samples (21%). Of these, nine were of good enough quality and length to be matched to mtDNA haplotypes previously described in Mirimin et al. (2011). Table 2 shows summary genetic diversity statistics of a 540 bp fragment of the mtDNA control region in the 3 putative populations, as identified by the microsatellite assignment described above. Among the total of 140 individuals for which mtDNA data was available, 15 distinct haplotypes were identified, including the 14 haplotypes described in previous studies (Mirimin et al. 2011), as well as one new haplotype (Ire15; Individual ID 40) from a dead stranded animal that was assigned to the offshore population with microsatellite data (see data in Table S5). The haplotype diversity of the inshore individuals was considerably lower, with only two haplotypes (Ire1, Ire2) found within the Shannon population and three haplotypes in the other coastal (Connemara, Mayo, Donegal) population (Table 2). The environmental samples collected at Brandon Point (Co. Kerry), outside the Lower Shannon SAC, matched either haplotype Ire1 or Ire2, which are the dominant haplotypes in the Shannon populations, further suggesting that the animals present at the time of sampling are likely to belong to the Shannon population. The only solitary dolphin that produced a consistent mtDNA sequence (Dusty) showed haplotype variant Ire2. As for population differentiation, the AMOVA analysis showed that variation was partitioned at 40% among populations and 60% within populations, with pairwise F_ST_ tests showing significant differentiation between the offshore and the Shannon (*F*_ST_ = 0.41, *p* < 0.01) and Connemara‐Mayo‐Donegal (*F*_ST_ = 0.23, *p* < 0.0001) coastal populations, as well as between the two coastal populations (*F*_ST_ = 0.55, *p* < 0.05), showing similar results to previous studies while using a larger sample size (Mirimin et al. 2011).

**TABLE 2:**
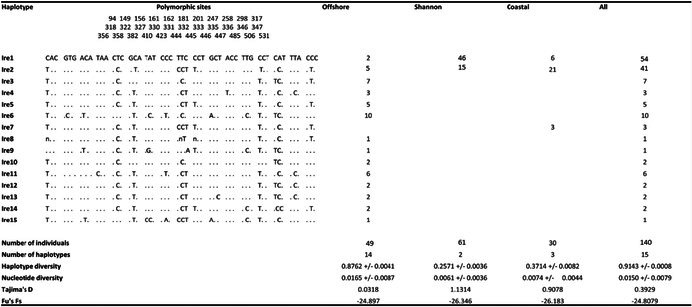
Mitochondrial DNA control region diversity in the three Irish populations as well as overall.

## 4 Discussion

The present study provides the most comprehensive population genetic analysis of bottlenose dolphins in Irish waters. Besides confirming the existence of 3 genetically distinct populations, as previously reported in Mirimin et al. (2011), this study showed how least invasive sampling methods such as eDNA from sea water samples can be viable approaches to obtain intra‐specific genetic data. The larger sample size analysed in this study allowed the discovery of a new mtDNA haplotype, while the targeted sampling of free‐ranging dolphins in Brandon Bay (Co. Kerry) provided further evidence supporting the need to further expand the boundaries of the Lower Shannon SAC.

### 4.1 Integration of Least Invasive DNA Samples

To date, studies using eDNA approaches to characterise intraspecific genetic variation in marine vertebrates have been limited to sequencing regions located in the mitochondrial genome (Sigsgaard et al. 2020; Elbrecht et al. 2018; Parsons et al. 2018; Weitemier et al. 2021; Bennington et al. 2024; Parsons et al. 2024). This is largely due to the higher availability of mtDNA in eDNA samples (multiple copies per cell) and the presence of hypervariable regions that can be used to determine haplotypes. In our study, we were able to obtain mtDNA sequences matching *Tursiops truncatus* in 11 out of 52 (21%) samples, of which 6 from eDNA from sea water. This relatively modest result could be due to the length of the region targeted (700 bp), as similar studies targeting a smaller fragment achieved more favourable results (Bennington et al. 2024; Székely et al. 2021). Nonetheless, sequencing allowed confirmation of species presence and, most importantly, all haplotypes generated from least invasive sampling matched haplotypes that are commonly known in the sampled areas, also aligning with population assignment results using microsatellite data.

Several studies have shown that intraspecific genetic data can be obtained from environmental samples, especially in freshwater systems (Stepien et al. 2019; Andres et al. 2021, 2023) and even in snow tracks (Hellström et al. 2023; De Barba et al. 2024), whereas this can be more challenging in marine ecosystems, where sampling must be targeted to ensure individual DNA is isolated, such as in marine mammals blow sampling (O'Mahony et al. 2024). The rate of success in amplification of microsatellite loci varied from sample to sample, with samples that contained visible faeces or sloughed skin performing better than sea water samples not collected in close proximity to individuals. Samples collected from least invasive methods (especially eDNA from sea water samples) required a higher level of scrutiny and curation to ensure that no false positive results or artefacts were included in the analysis. When working with a low number of loci per sample, undetected false alleles can have a large effect on subsequent analysis, such as population assignment (Gagneux et al. 1997; Bradley and Vigilant 2002). In the present study, prior knowledge of expected genetic variants and knowledge of amplification patterns (i.e., peak patterns) allowed the curation of microsatellite genotypes obtained from least invasive and eDNA samples, hence reducing the risk of inclusion of spurious results. As the microsatellite primers vary in their species‐specificity (as they were originally designed for specificity within the target species' genome as opposed to the multi‐taxa context of eDNA samples), it is theoretically possible that DNA from other cetaceans could generate alleles of the expected size and frequency and thus are indistinguishable from true bottlenose dolphin alleles. Nevertheless, mtDNA sequencing did not result in the detection of any other cetacean species aside from bottlenose dolphin in the samples used for microsatellite analysis, thus while it is still a possibility we believe that non‐specific amplification was negligible in this study. Another complication arising from working with microsatellite data of eDNA origin is the possible presence of multiple individuals within the sample. This could in‐turn generate composite genotypes, were the genotype derived from one sample derives from multiple individuals. This is likely to have occurred in the eDNA samples collected at Brandon Point where multiple individuals were bow riding in proximity to the boat during sampling. Thus, to overcome such challenge, future sampling efforts should focus on ensuring that samples collected using least invasive methods should ensure proximity to the individual being targeted so to limit the risk of mixing eDNA from multiple individuals in a single sample.

A similar discussion on specificity and individual composition can be applied to the sex markers results. As the sex primers for the target genes (SRY and ZFX) have the potential to amplify all cetaceans and all mammals respectively, it is difficult to exclude with certainty that some false results were included. This is potentially more probable in the case of the eDNA samples collected in presence of the solitary individuals, as these samples collected in coastal habitats are likely to include other mammal DNA, such as domestic animals, grey seal, otter, and human. However, the sex of the solitary individuals included in this study was already known, and all the successfully amplified samples collected in presence of said animals indicated a match between the visual and molecular results, suggesting a low level of false positive results. This challenge could be addressed in future studies by developing species‐specific molecular sexing markers to be used in conjunction with targeted least‐invasive sampling of individuals.

The present study showed that least invasive sampling (including eDNA from sea water) approaches are a promising tool for obtaining intra‐specific information from free‐ranging bottlenose dolphins, especially if obtained in close proximity to individuals. This can be facilitated by new emerging technologies such as Unmanned Aerial Vehicles (UAVs) (Arranz et al. 2024; O'Mahony et al. 2024; Neveceralova et al. 2025). Secondly, primers of newly developed genetic markers (or even existing markers) should be designed ensuring high specificity to the target species and/or the intra‐specific variant of interest. Specificity at the inter‐specific level is already commonly achieved in eDNA studies, especially when using qPCR assays (Langlois et al. 2021; Thalinger et al. 2021), whereas assays targeting variation at the intra‐specific level are increasingly being developed (e.g., Zanovello et al. 2024). Alternatively, but not exclusively, metabarcoding analysis of Amplicon Sequence Variants (ASV) could be used to query variation at the intra‐specific level (Elbrecht et al. 2018; Tsuji et al. 2020; Weitemier et al. 2021), provided that the markers are species specific. While this study showed that nuclear DNA can be obtained from eDNA samples and used at the intraspecific level, the lack of reference DNA data for homologous nuclear DNA regions in non‐target taxa will remain one of the biggest gaps to fill before nuclear markers are routinely employed in eDNA studies. Finally, despite the challenges outlined here, the use of composite genotypes (i.e., multi‐individual genotypes) is worth further consideration as it could inspire the development of novel metrics of population structure identification whereby population‐specific composite genotypes (as opposed to individual genotypes) could be defined for eDNA‐based analysis. The development of eDNA‐based sampling strategies would expand sample acquisition opportunities and greatly enhance the amount of genetic data that can be obtained from species of conservation concern, without the need to require special permits and/or harming the animals. Despite the limitations, the present study shows that eDNA data is a promising approach for the study of population structure of free‐ranging marine mammals.

### 4.2 Using Population Genetic Data for the Management of Irish Coastal Bottlenose Dolphin Populations

Levels of genetic diversity varied between the three delineated populations, with the putative offshore population showing highest levels of both nuclear and mitochondrial diversity. The two coastal populations (Shannon and Connemara‐Mayo‐Donegal) showed lower levels of polymorphism, although in different proportions across different loci. This is in agreement with previous studies of bottlenose dolphins in the North Atlantic, which reported that offshore populations show higher levels of genetic diversity compared to coastal populations (Hoelzel et al. 1998; Natoli et al. 2005; Nichols et al. 2007; Quérouil et al. 2007; Louis et al. 2014; Nykänen et al. 2018). While uncommon, strong genetic differentiation at small geographic scales can be found in marine mammals (Palumbi 1994), especially in coastal populations that display a degree of residency all year round (Mirimin et al. 2011). This was further confirmed in the present study, whereby even in Irish coastal waters multiple populations can co‐exist such as the Shannon and the Connemara‐Mayo‐Donegal populations, even though ongoing photo‐identification demonstrates that they can overlap in distribution and occurrence (IWDG unpublished data). This is also supported by two stranded individuals in Co. Kerry and Clare that clustered in the Connemara‐Mayo‐Donegal population, hence suggesting that interbreeding may be ongoing but may be limited thus maintaining these populations as genetically distinct. Social structure analyses using long‐term photo‐identification data confirm that the two coastal populations are socially distinct (O'Brien et al. 2009, Nykänen et al. 2018). To this day, no distinction in morphology, diet or niche specialisation has been recognised between these two coastal populations and thus reasons for this isolation should be explored in future studies.

Populations characterised by a small effective population size (Ne) and a high degree of isolation, such as the case of the bottlenose dolphins inhabiting the Lower River Shannon SAC, are especially vulnerable to any environmental or anthropogenic stressors. Habitat degradation and other stressors could bring the population to face extinction in the next 50 years (Blázquez et al. 2020), as occurred in the Humber estuary (East England) where a genetically differentiated population of bottlenose dolphins became extinct, and the estuary was never repopulated (Nichols et al. 2007). This emphasises the importance of the Lower River Shannon SAC status as a means to maintain the Shannon population at Favourable Conservation Status. Results from this study indicate that the dolphins belonging to the Shannon population often venture outside of the Lower River Shannon SAC, as for the two individuals biopsied and the seven eDNA samples collected in Brandon Bay (Co. Kerry). This is in agreement with a recent acoustic study (Charish et al. 2021) that shows consistent bottlenose dolphin presence and foraging activity in Brandon Bay. Levesque et al. (2016) using photo‐identification showed bottlenose dolphins foraging in Brandon Bay belong to the Shannon population. These studies and the current one clearly indicate that Brandon Bay is a critical area for the Shannon dolphin population and, as such, the boundaries of the Lower River Shannon SAC should be expanded to include this important habitat. There is some evidence to suggest that some individuals from the Shannon population are spending less time in the estuary and may also demonstrate permanent emigration (Ludwig et al. 2021). Bottlenose dolphins stranded up to 70 km north of the Lower River Shannon boundary and 45 km west in Brandon Bay, which is further outside the boundary than reported in an earlier study by Mirimin et al. (2011) support this range expansion and that Shannon bottlenose dolphins may be more mobile than previously thought. Protecting foraging hotpots outside the Shannon Estuary could be essential to ensure the long‐term survival of the Shannon population, thus this study adds to an increasing body of evidence urging for reconsideration of the current SAC boundaries.

The management and protection of a highly mobile animal can be challenging (Game et al. 2009; Breen et al. 2015; Nykänen, Kaschner, et al. 2019; Nykänen, Louis, et al. 2019). Findings from the present study (14 new biopsy samples) indicate that individuals from the Coastal population range from Galway Bay to Donegal Bay, covering over 400 km of coastline and much further (O'Brien et al. 2009; Robinson et al. 2012). These results confirm the mobile nature of this bottlenose dolphin population, which ranges far outside the West Connacht Coast SAC which is designated for their protection. In recent years, bottlenose dolphins from this population as determined by photo‐id have been frequently re‐sighted in Northern Ireland (Antrim Coast), Dublin Bay, Cork Harbour and Dingle Bay, thus indicating that these individuals' range encompasses the entirety of the Irish coastline. Long distance movements by coastal bottlenose dolphins between the UK (Scotland and Wales) and Irish waters (Robinson et al. 2012) have also been recorded, suggesting the incredible range of movements of the Coastal population. A large population of bottlenose dolphins inhabiting Northeast Atlantic coastal waters was identified by Louis et al. (2014), in which dolphins from Moray Firth (Scotland), Cardigan Bay (Wales) and Western Ireland all clustered together when examined with a large set of microsatellite markers. This shapes the idea of a large coastal population of bottlenose dolphins comprised of residents, which are localised to certain areas, and transient animals, which migrate seasonally into and out of key areas. The large‐scale movement of these transient dolphins makes it difficult to define singular conservation units, especially since their movements bring them across international waters. A network of SACs with migration corridors could be an option as an effective approach when managing the conservation of the Connemara‐Mayo‐Donegal coastal population. A similar strategy has been proven successful for the protection of marine mammals, sharks, and marine turtles in Australia thanks to the establishment of the Commonwealth Marine Reserves (Pendoley et al. 2014). Following the designation of a further five SACs with bottlenose dolphin as a qualifying interest including three offshore sites (Porcupine Bank Canyon SAC, Southwest Porcupine Bank SAC and Belgica Mound Province SAC) and two coastal sites (St Johns Point SAC and Hook Head SAC), it would be of interest to obtain samples from these sites to determine which population utilise these habitats.

### 4.3 Solitary Social Individuals

Solitary bottlenose dolphins that tend to stay in a very small discrete area offered a great opportunity to explore least invasive sample acquisition for intra‐specific eDNA analysis and hence, albeit indirectly, attempt to explain such behaviour. In our study, all the solitary social individuals were assigned to the Offshore population, except for Fungie, which showed an intermediate genotype (Shannon/Offshore). Furthermore, all but Dusty were confirmed to be males. Delphinids are often characterised by a strong social structure, which is likely the result of benefits gained through cooperation, in terms of access to resources (food) and mates (reproduction) as well as predator avoidance. Even though it is not the norm for this species, it is possible that solitary dolphins may become independent from their source population for whatever reason, and they may find enough resources in a given area to survive, even if this limits their chances of reproduction, as is the case in other solitary mammal species (Makuya and Schradin 2024). Whether this is the result of an active choice of the individual or a consequence of being “expelled” from a social unit remains a mystery; however, interestingly, findings from the present study showed that there was a strong sex bias in stranded dolphins belonging to the Offshore ecotype population (twice as many males as females), suggesting that males may be more likely to become isolated and independent from their source population. This is in contrast to coastal and resident populations that may have stronger social bonds and may be more reluctant to expand their range, which in turn would make them more vulnerable to local habitat degradation, as may be the case of the coastal populations found in Irish waters.

## Author Contributions

**Lorenzo De Bonis:** conceptualization (lead), data curation (lead), formal analysis (lead), investigation (equal), methodology (equal), validation (equal), visualization (equal), writing – original draft (lead), writing – review and editing (lead). **Bogna Griffin:** conceptualization (equal), data curation (equal), investigation (equal), methodology (equal), writing – original draft (equal), writing – review and editing (equal). **Sean O'Callaghan:** conceptualization (equal), data curation (equal), methodology (equal), writing – original draft (equal), writing – review and editing (equal). **Mags Daly:** writing – original draft (equal), writing – review and editing (equal). **Jade Maes:** conceptualization (equal), data curation (equal), formal analysis (equal), methodology (equal), writing – original draft (equal), writing – review and editing (equal). **Simon Berrow:** conceptualization (lead), funding acquisition (lead), project administration (equal), resources (lead), supervision (equal), writing – original draft (equal), writing – review and editing (equal). **Luca Mirimin:** conceptualization (lead), data curation (equal), funding acquisition (lead), investigation (equal), methodology (equal), project administration (lead), resources (equal), supervision (lead), validation (equal), visualization (equal), writing – original draft (lead), writing – review and editing (equal).

## Conflicts of Interest

The authors declare no conflicts of interest.

## Supporting information


**Figure S1:** Maps of the Lower River Shannon SAC (Figure S1a) and the West Connacht Coast SAC (Figure S1b). Further details of statutory maps available on https://www.npws.ie/protected‐sites/sac/002165 and https://www.npws.ie/protected‐sites/sac/002998, respectively.

**Figure S2:** Power analysis to investigate the minimum number of successfully genotyped loci to correctly assign a sample to its population of origin (cluster K). A cumulation curve is shown for a simulation of 140 separate STRUCTURE runs, using 100 samples that were successfully genotyped at all 15 markers. In each run, a random combination of markers had missing data across all samples, 10 combinations for each possible number of no‐data markers (1–14), and the result was then compared to the run with all 15 markers.

**Figure S3:** Results of structure harvester analysis of 10 Markov Chains Monte Carlo (MCMC) runs carried out for each of six *K*‐values (1–6), using the admixture model and correlated allele frequencies among putative populations. The parameters chosen for the runs where 100,000 (burn in) and 1,000,000 MCMC repetitions. The value of *L*(*K*) shows a clear rising trend up to *K* = 3 where the trend is inverted. The value of Delta K shows a steep decreasing trend until *K* = 3 where it plateaus.

**Table S1:** Metadata associated to samples used int. his study. Lines highlighted in green indicate individuals that were used in Mirimin et al. (2011) and re‐processed in this study to enable the merging of previous with new datasets.

**Table S2:** List of markers used in the current study, showing multiplex combinations, number of alleles and size ranges (microsatellite only) and non‐specificity results following a BLAST search.

**Table S3:** Microsatellite genotypes of DNA from tissue samples.?: missing data. Uncertain/alternative alleles are shown in parentheses.

**Table S4:** Levels of genetic diversity at 15 microsatellite markers across all three populations of bottlenose dolphins found in Irish waters. Na: number of alleles; Np: number of private alleles; I: Shannon's Index; Ho: observed heterozygosity; He: expected heterozygosity; F: Fixation Index.

**Table S5:** Mitochondrial DNA sequence data and associated metadata.

## Data Availability

The data that support the findings of this study are available in the Supporting Information of this article.
