# Supplementary material for: Enhancing Intra‐Specific Population Genetic Studies of Bottlenose Dolphins ( Tursiops truncatus ) in Irish Waters by Integrating Least Invasive Sampling and Environmental DNA (eDNA)
Source: Ecol Evol. 2026 Aug 14;16(8):e74035. doi: 10.1002/ece3.74035 (PMC13476162; doi:10.1002/ece3.74035)
Supplement: Supplementary file 1 [file ECE3-16-e74035-s001.docx]

**a)**

**b)**

**Figure S1**. Maps of the Lower River Shannon SAC (Figure S1a) and the West Connacht Coast SAC (Figure S1b). Further details of statutory maps available on <https://www.npws.ie/protected-sites/sac/002165> and <https://www.npws.ie/protected-sites/sac/002998>, respectively.
