# Supplementary material for: Enhancing Intra‐Specific Population Genetic Studies of Bottlenose Dolphins ( Tursiops truncatus ) in Irish Waters by Integrating Least Invasive Sampling and Environmental DNA (eDNA)
Source: Ecol Evol. 2026 Aug 14;16(8):e74035. doi: 10.1002/ece3.74035 (PMC13476162; doi:10.1002/ece3.74035)
Supplement: Supplementary file 2 [file ECE3-16-e74035-s002.docx]

**Figure S3** Results of structure harvester analysis of 10 Markov Chains Monte Carlo (MCMC) runs carried out for each of six K-values (1–6), using the admixture model and correlated allele frequencies among putative populations. The parameters chosen for the runs where 100 000 (burn in) and 1 000 000 MCMC repetitions. The value of L(K) shows a clear rising trend up to K = 3 where the trend is inverted. The value of Delta K shows a steep decreasing trend until K = 3 where it plateaus.
