# Supplementary material for: Enhancing Intra‐Specific Population Genetic Studies of Bottlenose Dolphins ( Tursiops truncatus ) in Irish Waters by Integrating Least Invasive Sampling and Environmental DNA (eDNA)
Source: Ecol Evol. 2026 Aug 14;16(8):e74035. doi: 10.1002/ece3.74035 (PMC13476162; doi:10.1002/ece3.74035)
Supplement: Supplementary file 3 [file ECE3-16-e74035-s003.docx]

**Table S4** Levels of genetic diversity at 15 microsatellite markers across all three populations of bottlenose dolphins found in Irish waters. Na: number of alleles; Np: number of private alleles; I: Shannon’s Index; Ho: observed heterozygosity; He: expected heterozygosity; F: Fixation Index.

**Np**
