# Supplementary material for: Enhancing Intra‐Specific Population Genetic Studies of Bottlenose Dolphins ( Tursiops truncatus ) in Irish Waters by Integrating Least Invasive Sampling and Environmental DNA (eDNA)
Source: Ecol Evol. 2026 Aug 14;16(8):e74035. doi: 10.1002/ece3.74035 (PMC13476162; doi:10.1002/ece3.74035)
Supplement: Supplementary file 4 [file ECE3-16-e74035-s006.docx]

**Figure S2** Power analysis to investigate the minimum number of successfully genotyped loci to correctly assign a sample to its population of origin (cluster K). A cumulation curve is shown for a simulation of 140 separate STRUCTURE runs, using 100 samples that were successfully genotyped at all 15 markers. In each run, a random combination of markers had missing data across all samples, 10 combinations for each possible number of no-data markers (1 to 14), and the result was then compared to the run with all 15 markers.
